# Review article: Current status and future directions of ingestible electronic devices in gastroenterology

**DOI:** 10.1111/apt.17844

**Published:** 2024-01-02

**Authors:** Phoebe A. Thwaites, Chu K. Yao, Emma P. Halmos, Jane G. Muir, Rebecca E. Burgell, Kyle J. Berean, Kourosh Kalantar‐zadeh, Peter R. Gibson

**Affiliations:** ^1^ Department of Gastroenterology, Central Clinical School Monash University and Alfred Health Melbourne Victoria Australia; ^2^ Atmo Biosciences Melbourne Victoria Australia; ^3^ School of Engineering, RMIT University Melbourne Victoria Australia; ^4^ Faculty of Engineering, School of Chemical and Biomolecular Engineering The University of Sydney Camperdown New South Wales Australia

## Abstract

**Background:**

Advances in microelectronics have greatly expanded the capabilities and clinical potential of ingestible electronic devices.

**Aim:**

To provide an overview of the structure and potential impact of ingestible devices in development that are relevant to the gastrointestinal tract.

**Methods:**

We performed a detailed literature search to inform this narrative review.

**Results:**

Technical success of ingestible electronic devices relies on the ability to miniaturise the microelectronic circuits, sensors and components for interventional functions while being sufficiently powered to fulfil the intended function. These devices offer the advantages of being convenient and minimally invasive, with real‐time assessment often possible and with minimal interference to normal physiology. Safety has not been a limitation, but defining and controlling device location in the gastrointestinal tract remains challenging. The success of capsule endoscopy has buoyed enthusiasm for the concepts, but few ingestible devices have reached clinical practice to date, partly due to the novelty of the information they provide and also due to the challenges of adding this novel technology to established clinical paradigms. Nonetheless, with ongoing technological advancement and as understanding of their potential impact emerges, acceptance of such technology will grow. These devices have the capacity to provide unique insight into gastrointestinal physiology and pathophysiology. Interventional functions, such as sampling of tissue or luminal contents and delivery of therapies, may further enhance their ability to sharpen gastroenterological diagnoses, monitoring and treatment.

**Conclusions:**

The development of miniaturised ingestible microelectronic‐based devices offers exciting prospects for enhancing gastroenterological research and the delivery of personalised, point‐of‐care medicine.

## Graphical abstract

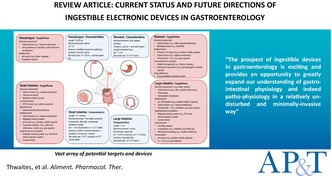

Illustrates the typical characteristics of each section of the gastrointestinal tract from oesophagus to large intestine (light blue) and many potential targets for ingestible electronic device technology (peach). H_2_, Hydrogen gas; cfu, colony forming unit; CO_2_, carbon dioxide.

## 1 INTRODUCTION

Ingestible electronic microsystems containing sensors that can capture and transmit an array of biological data telemetrically, and/or deliver interventional functions represent a burgeoning area of science. These ingestible electronic microsystems can also contain sampling and drug‐release components. Until recently, the research and development for commercialisation of these ingestible devices have been tempered by technological challenges related to power supply, biocompatibility, device miniaturisation and the full demonstration of clinical relevance and economic viability. Considerable advances in many of these areas in recent times have enabled the design of a number of devices with screening, therapeutic and diagnostic capabilities. Altogether, such technologies have the potential to greatly alter historic paradigms of healthcare assessment and delivery, introducing a new era of personalised, digital medicine, especially in the field of gastroenterology.

Ingestible devices are attractive tools for the inspection and assessment of the gastrointestinal tract due to its extended length and relative inaccessibility, allowing longitudinal analysis in real‐time, in an ambulatory setting and possibly with minimal patient inconvenience. Understanding the complex interplay between microbiome, host and dietary substrate and the locoregional variation of structure and function of the gastrointestinal tract is pivotal in the correct interpretation of gastrointestinal tract physiology in health and disease.^1^ Ingestible devices are likely to play an increasing role in this regard. There are a myriad of new devices in the pipeline. In light of the limited awareness of this rapidly expanding field, which is likely to infiltrate many areas of clinical practice in the not‐too‐distant future, the current narrative review aims to provide a comprehensive overview for the clinician, by exploring the key components of ingestible electronic devices and introducing some of the novel devices in the research and development or commercial pipeline. It will also explore the current challenges associated with the introduction of this technology into clinical practice and its future directions.

## 2 METHODOLOGY

In order to perform this narrative review, published literature, including those in abstract form, were systematically searched using PubMed, ProQuest, OVID and Google Scholar using keywords, that included ingestible, electronic devices, telemetric devices, capsules and sensors. Each subsection was additionally explored using targeted searching, for example, ‘ingestible electronic device AND microbiome’. Abstracts were appraised and relevant articles were then reviewed. Additional studies were located via cross‐referencing and via searching news reports/press releases and websites of technology companies. Studies in paediatric cohorts were not included.

## 3 A PRIMER FOR WHAT IS REQUIRED TO CREATE AN INGESTIBLE ELECTRONIC DEVICE

**FIGURE 1:**
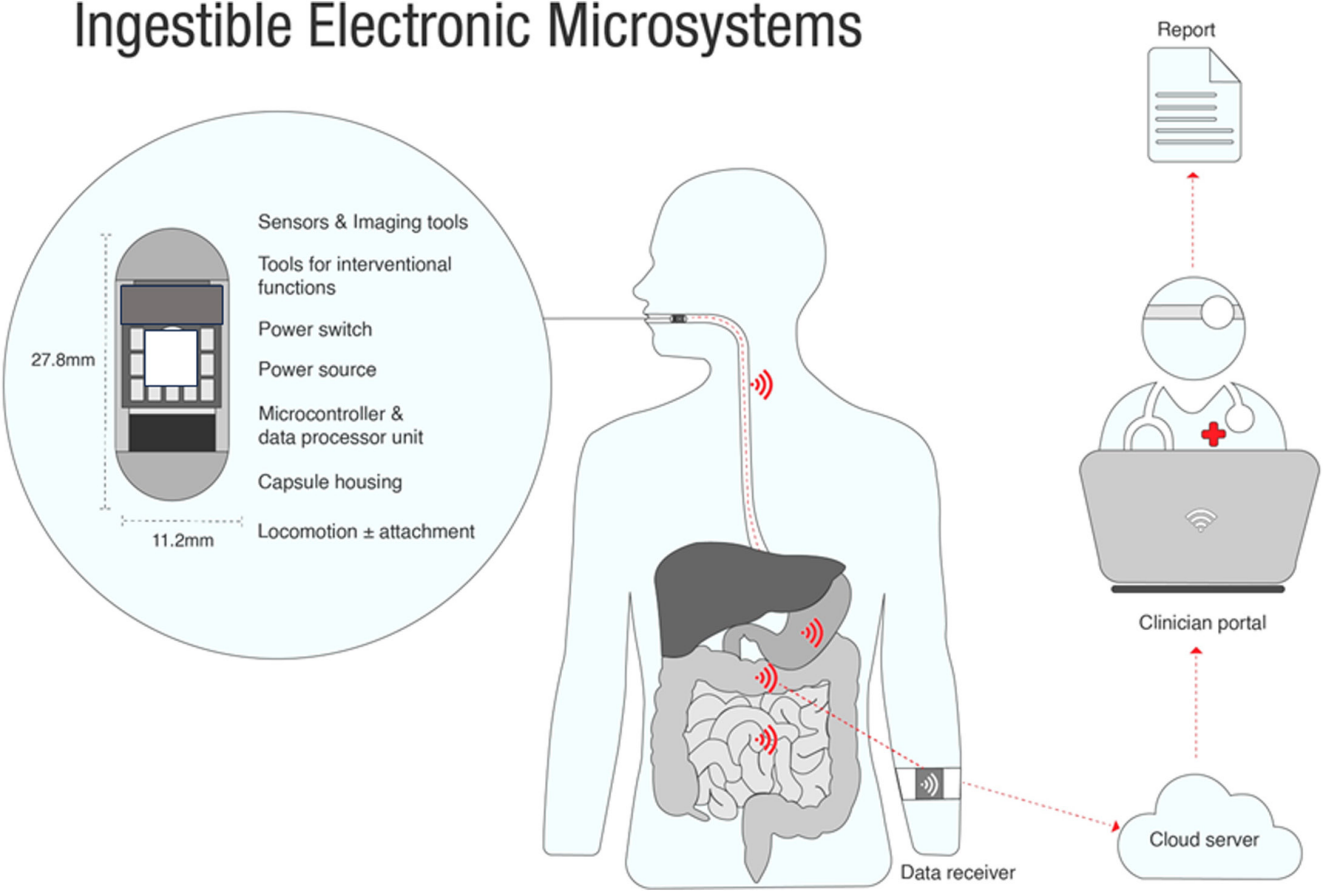
An overview of an ingestible electronic microsystem, including the ingestible capsule and its various internal components (which vary according to a capsule's specific function), wearable data receiver and clinician portal and personalised report. Modified from Alazemi and Iqbal.^2^

A description of components is needed to enable an understanding of issues such as the device's capabilities, risk, cost and privacy. Typical components are illustrated in Figure 1.

- ***Sensors and imaging tools***: These have been designed to interact with the luminal microenvironment and measure biological information. Various sensors exist, including optical (camera), thermal, pH, pressure, semiconducting, fluorescence, spectrometric, electrochemical, acoustic and sonographic electronic conversion tools, which ultimately define the function of the device and is a major determinant of the device's battery requirements.
- ***Power source***: The ingestible device requires a power source, which may be battery operated, or more recently, battery‐free. The capacity of the power source must be high enough to meet the needs of the microelectronic circuit and specific sensors and allow for sufficient operational time to complete the intended test. In battery‐operated devices, important considerations include the type of battery used, the amount of heat it generates as well as its size, the latter of which dictates the ingestible device's overall size. For example, silver oxide batteries are small and do not produce excessive current during short‐circuiting, but have a low power‐to‐weight ratio. In comparison, lithium batteries have better performance, but generate heat and carry the risk of thermal runaway, a faulty cycle catalysed by increased temperature that risks battery combustion.^3^, ^4^ Non‐battery sources of power, such as electromagnetic, piezoelectric (using kinetic energy) and triboelectric (using friction) have also been investigated in capsules and recently, there has been a focus on the development of biofuel cells, which generate energy via enzymatic or microbe‐related biochemical reactions.^5^, ^6^ Biofuel cells, such as the glucose biofuel cell, are biocompatible and do not generate much heat, but are expensive and their use has been limited by short lifespan, low power output and technological inconsistencies.^6^
- ***Power switch***: In order to conserve battery life, a switch is required to activate the electronic device prior to ingestion. This is often a magnetic switch, known as a reed switch.
- ***Microcontroller and data processor unit***: Microcontrollers are crucial for all operational aspects of ingestible devices, including how and when to drive sensors and read the sensor output signals, storing and manipulating raw data for further interpretation and transmission and triggering mechanical events of the device such as opening cavities for therapeutic drug delivery or sampling.^7^
- ***Data collection and transmission***: A data receiver with a receiving antenna system and battery pack allows the data to be received from the electronic device. Wide bandwidth telecommunication is essential to allow this data transmission to occur. For example, 433 MHz is a common frequency used by biological devices.^8^, ^9^ Bluetooth® is an alternative way in which data may be transferred.^9^ Of critical importance, data transferred must be protected to ensure patient privacy. If the transfer of data is not possible, such as when samples or other data are collected and stored within the device, such as with CapsoCam Plus capsule endoscopy, the data may be retrieved upon collection of the device post‐excretion.^10^, ^11^
- ***Capsule housing (cladding)***: The individual components of the device are housed in an inert, biocompatible, pill‐shaped cladding resistant to damage from intestinal secretions (such as acid and enzymes) and microbial actions.^1^ Its size and shape are ultimately dictated by the components within, but ideally should be of a size amenable to swallowing and with minimal risk of retention. A typical size is less than 13 mm diameter and 34 mm length. A semipermeable membrane may be present to allow interaction between the sensor component and the lumen or to allow sampling to occur.
- ***Workstation with software program***: Data obtained may be reviewed in real‐time or upon completion of the study. Dedicated software will allow data reporting and may include an automated computer‐generated reporting option. Devices may capture several hours of data for review, and computer‐assisted reviewing algorithms may be present to reduce viewing times and assist diagnosis. These may use artificial intelligence and machine learning (e.g. convolutional neural network, decision trees or clustering algorithms).^12^

## 4 MOVING THE CAPSULE THROUGH THE GASTROINTESTINAL TRACT

Movement of the device through the gastrointestinal tract may be uncontrolled, or passive, or controlled through active manipulation. Similarly, movement can be inhibited. *Passive locomotion* is influenced by gravity and peristalsis of the bowel, which is variable and relatively unpredictable. In the presence of altered motility, this passive movement may result in limited visualisation of the bowel mucosa (and any lesion of interest) or lead to excessively slow or fast transit, reducing the device's diagnostic accuracy. Opportunity for therapeutic intervention may also be limited due to the difficulties with targeting a specific lesion or site in the gastrointestinal tract for treatment, sampling or drug delivery. *Active manipulation of locomotion* can be achieved through capsule design, which is mostly experimental, and includes capsule legs,^13^, ^14^ paddles^15^ and inchworm‐like manoeuvrability^16^ or can be externally controlled such as through the use of magnetic fields and/or external tethering such as string.^17^, ^18^ Active locomotion capabilities allow more intentional and targeted capsule movement to be achieved. *Inhibition of locomotion* may enable the device and its sensor to spend a prolonged period of time at a specific site in the gut, such as the Bravo™ pH capsule, which is endoscopically clipped to the distal oesophageal mucosa.^19^ Alternatively, the physical properties of the device may be designed in such a way that the effects of peristalsis are reduced, allowing increased gastric or intestinal residency time. For example, a device of low density was demonstrated to increase gastric residence time in a gastric physiology study in pigs,^20^ although such an approach might be challenging to safely apply in humans. This concept has been discussed in greater detail previously.^21^

## 5 THE LANDSCAPE OF INGESTIBLE SENSORS IN GASTROENTEROLOGY

A wide spectrum of sensing tools have been applied to the human gastrointestinal tract measuring various aspects of gastrointestinal function and/or structure. To date, few have reached routine clinical practice, but several have promising development programs. Examples are outlined below.

### 5.1 Imaging the gastrointestinal tract

The majority of imaging tools currently used in clinical practice have the potential to be adapted, miniaturised and incorporated into ingestible device technology. Examples are included in the following.

- ***Direct imaging***: Direct imaging using capsule endoscopy represents a significant portion of the market for ingestible sensors. Video capsule endoscopy was first described in 2000 with the PillCam® (originally known as the M2A capsule, mouth to anus) first receiving FDA approval in 2001.^22^, ^23^ The images obtained from the most recent iterations of ingestible capsules with optical cameras are now of comparable resolution to fibre‐optic endoscopy. Details of how many of the technical challenges and obstacles have been overcome have been reviewed elsewhere.^4^

There are several video capsule endoscopy systems commercially available across the globe, including, the PillCam (Medtronic, Minneapolis, USA), which has various iterations specifically designed for endoluminal assessment of the oesophagus (e.g. PillCam ESO/Upper GI), small intestine (e.g. PillCam SB3) and large intestine (e.g. COLON2)^24^, ^25^; the EndoCapsule (Olympus, Tokyo, Japan)^26^; MiroCam (IntroMedic, Seoul, Korea)^27^; CapsoCam (Plus) (CapsoVision, Saratoga, USA)^11^, ^28^ and the OMOM capsule (Jianshan Science and Technology (Group) Co., Ltd., Chongqing, China).^29^ Wireless capsule systems continue to be refined.^30^ The commercially available capsules vary slightly in design, both in physical capsule characteristics and by function, including the lens field of view, how the images are captured, the frame rate of image acquisition, battery life, data viewing system and the need (or not) for post‐excretion capsule retrieval.^11^, ^31^

Capsule endoscopy technology is now considered the gold standard in the work‐up of obscure gastrointestinal bleeding and accounts for approximately two thirds of the capsule studies performed.^31^ The European Society of Gastrointestinal Endoscopy also reference the use of capsule endoscopy in various other scenarios, such as *suspected* small bowel Crohn's disease (in the absence of obstructive symptoms or obvious stricturing disease and negative ileocolonoscopy) and in certain clinical settings for surveillance of polyposis syndromes.^32^, ^33^ In *established* Crohn's disease, prior administration of a patency capsule may be appropriate to assess the risk of capsule retention.^32^ Other potential indications include NSAID enteropathy, after a recommended minimum 4‐week cessation,^33^ graft‐versus‐host disease and coeliac disease in certain clinical scenarios.^34^, ^35^, ^36^ Since gastric exit and caecal entry are readily identified by distinct imaging features, regional gastrointestinal transit times can also be calculated using the time of transit through these landmarks. Video capsule endoscopy has also been used to assess motility patterns in the small bowel^37^ and stomach.^38^ The failure rates of these devices relate largely to the passive nature of their transit, and are inherently at risk of missing lesions, depending on the direction and field of view of the lens, the lesion location and size, rate of intestinal transit and the bowel preparation.^32^, ^39^ Failure rates will also be influenced by the primary indication for the capsule endoscopy and the timing of administration of the capsule, for example, the diagnostic yield for capsule endoscopy in obscure gastrointestinal bleeding is higher if performed early (e.g. within 48–72 h of the bleeding event).^32^, ^39^

More advanced capsule endoscopy systems, including the OMOM capsule system, have duplex data transmission, which means that real‐time monitoring is possible with the ability to adjust the activity of the device, such as the image capture frequency and other image format characteristics (e.g. flash intensity and white balance) using a workstation, thereby enhancing small bowel examination.^29^

A number of magnetically‐controlled endoscopy systems have been developed since the original concept was proposed in 2006.^18^, ^38^, ^40^, ^41^, ^42^, ^43^ The magnetic control may be hand‐held, MRI‐based or robotic.^18^, ^44^ The use of a detachable string in conjunction with the magnetically controlled capsule endoscopy system (e.g. Ankon Technologies Co. Ltd, Shanghai, China) has been studied, to increase the assessment of the upper gastrointestinal tract, particularly the oesophagus.^18^, ^45^, ^46^ The application of magnetically‐controlled capsules in gastric disease assessment has been reviewed in detail previously.^47^ Robotically‐assisted magnetic endoscopy systems have also been described, including those, in‐concept, which can be anally inserted and used in the assessment of colonic pathology.^48^, ^49^

- ***Optical sensors***: Reflection, absorption, transmission and scattering of light can be measured by optical sensors. An example of such sensors is the telemetric bleeding sensor (HemoPill, Ovesco Endoscopy Endoscopy AG, Tuebingen, Germany), where the ratio of red and violet light measured by an LED (light emitting diode) sensor can detect blood, in a non‐fasted patient, which has the potential to allow rapid assessment and triaging of patients presenting with possible upper gastrointestinal bleeding.^50^, ^51^ The obvious downside to this is that no intervention can be applied when bleeding is identified.
- ***Sonographic transducers***: Ultrasound capsule endoscopy (USCE) performs transmural imaging and both qualitative and quantitative assessment of the gastrointestinal tract wall, in contrast to optical imaging (e.g. video capsule endoscopy) which is generally limited to assessment of the mucosal surface.^52^ Micro‐ultrasound waves are high in frequency (>20 MHz) and provide shallow but high‐resolution imaging, which has been shown to correlate with histological findings.^53^, ^54^, ^55^ The therapeutic and interventional capabilities of these transducers have been evaluated in proof‐of‐concept studies and include applications such as ultrasound‐mediated targeted drug delivery. Transient permeabilisation of the mucosal membrane may be achieved using this technology enabling molecular diffusion.^56^, ^57^, ^58^ The SonoPill capsule system was being developed with diagnostic and therapeutic capabilities in mind, but has now been discontinued.^57^, ^59^ Research and development continues in this field and includes robotically‐controlled ultrasound capsule technology.^59^, ^60^
- ***X‐ray sensors***: Radiological assessment of the large intestine has been achieved using X‐ray technology, with short‐lived radioisotope emitting low‐dose X‐ray beams from an ingested device, equivalent to one chest X‐ray.^61^ The patients ingest a small volume of radiopaque contrast agent in lieu of bowel preparation. The position of the device can then be tracked using a 3D accelerometer and 3D magnetometer. The data obtained enable a three‐dimensional reconstruction of the colon to be achieved with certain polyps and other lesions identifiable depending on their size and morphology. There may be a role for a diagnostic capsule such as this in screening patients not willing or able to proceed in the first instance to a colonoscopy. However, the device itself does not have therapeutic capability, such as polypectomy, so may not obviate the need for colonoscopy.

### 5.2 Sensors of position and orientation

- ***Radiofrequency identification (RFID) sensors***: Devices containing elements capable of interacting with electromagnetic waves are able to be detected by radiofrequency and are the basis for the dissolvable patency capsules used to assess the risk of capsule retention.^62^
- ***Spatiotemporal analysis***: Spatiotemporal analysis of an ingestible device in the gastrointestinal tract is possible using magnetic tracking technology, in which position and angle coordinates are derived from electromagnetic fields. Position coordinates reflect the capsule's three‐dimensional spatial position, which allows transit time between two anatomical locations to be calculated. Angular coordinates, on the contrary, serve as a surrogate of intestinal contraction frequency, which is different in each region of the gastrointestinal tract. This area of development is very active with several prototypes described.^63^

The earliest system using this technology was the Magnet Tracking System (MTS‐1), comprising of a stationary detector for the patient to lie on, a small ingestible magnetic ‘pill’, a calibrated detection matrix with multiple magnetic field sensors (worn across the subject's abdomen) and software to analyse the output.^64^, ^65^, ^66^ By contrast, the more recent 3D transit electromagnetic system (Motilis Medica, SA, Lausanne, Switzerland) is an ambulatory system that uses a portable detector. The system has been used in the assessment of regional and whole gut transit and colorectum length, motility and colonic segmental transit time.^67^, ^68^, ^69^, ^70^, ^71^

An ambulatory system using electromagnetic coils, known as the iMAG system (ingestible microdevices for anatomical mapping of the gastrointestinal tract), is in the early stages of development. Such a system may have a potential role in the assessment of gastrointestinal transit and in combination with other devices to enable targeted delivery due to its localisation capability.

- ***Accelerometer***: This detects motion by measuring the rate of change of movement over time. These sensors are commonly marketed for health and lifestyle movement tracking and have been incorporated in several ingestible electronic devices for localisation purposes (e.g. Atmo gas‐sensing capsule) and to help measure movement artefact (e.g. 3D transit electromagnetic system).^72^, ^73^, ^74^, ^75^

### 5.3 Chemosensors

pH assessment has been used to assess various aspects of the gastrointestinal system.^19^, ^76^, ^77^ From the distal oesophagus, in the measurement of gastroesophageal reflux, to caecal fermentation in the large intestine. The Bravo catheter‐free pH monitoring system, for example, measures acid exposure for an extended period (>36 h in 89% of patients) after being endoscopically clipped to the distal oesophagus (Bravo, Medtronic/Synectics, USA).^19^ The device is well‐tolerated overall, although non‐cardiac chest pain is experienced at increased frequency compared with controls.^78^, ^79^ The OMOM pH capsule (Chongqing Jinshan Science and Technology Group Co., Ltd, Beijing, China) similarly, measures oesophageal acid exposure by monitoring pH for extended periods up to 96 h in duration. A magnetically‐held pH and impedance monitoring capsule device was reported in concept some time ago but does not appear to have progressed.^80^

The principal sensor in the SmartPill® WMC monitoring system is a pH sensor.^81^, ^82^, ^83^ The presence of predictable pH changes along the tract allows the capsule to be localised during its transit through the gastrointestinal tract. These changes include an abrupt and sustained rise in pH that occurs on transition from the acidic environment of the stomach to the bicarbonate‐rich environment of the duodenum (pH 5.9–6.8) and then the abrupt fall in pH from the alkaline ileum (pH ~7.3–7.7) to the acidic caecum/proximal ascending colon (pH 5.8–6.7), occurring secondary to the production of weak acids, such as short‐chain fatty acids, from carbohydrate fermentation.^84^, ^85^, ^86^, ^87^ The WMC was approved by the FDA in 2006 for evaluating gastric emptying and colonic transit times in suspected gastroparesis and slow‐transit constipation, respectively and it has also been used to evaluate colonic fermentation in the colon, but with less validation.^88^, ^89^, ^90^ In June 2023 it was announced that WMC production will be terminated.^91^ An alternative ingestible pH‐sensing capsule, which is low in cost, has recently been reported and further studies are awaited.^92^

Reactive oxygen species are able to be measured using electrochemical sensors and have been described in proof‐of‐concept recently, combined with a pH sensor to help localise the capsule within the gastrointestinal tract.^93^ It has been proposed as an alternative biomarker to assess inflammation, such as in inflammatory bowel disease.

### 5.4 Gas sensors

Gases are valuable biomarkers in the gut.^94^ The Atmo gas‐sensing capsule (Atmo Biosciences, Box Hill, Australia) is a novel ingestible device that houses analyte sensors capable of measuring hydrogen and carbon dioxide concentrations using thermal conductivity detectors, a multi‐gas detector via a volatile organic compound sensor and also measures temperature, capsule orientation and antennae efficiency.^95^, ^96^ Measurements are transmitted from the capsule at a frequency of 434 MHz to a patient‐worn data receiver and subsequently uploaded to a remote server via a mobile device application for analysis and review via a web portal. It has been demonstrated to assess major gastrointestinal landmarks accurately in healthy subjects and a pilot study of gastroparesis and slow transit constipation and is being validated in larger trials for transit assessment presently.^75^, ^97^ Analysis of gas profiles may also enable additional physiological processes to be described, including colonic fermentation^74^ and small intestinal bacterial overgrowth (SIBO).^98^ A prototype capsule system detecting hydrogen sulphide has also recently been described.^99^

### 5.5 Microbiological‐based sensors

Genetically modified probiotic sensor bacteria can be used to help detect specific analytes in the gastrointestinal tract lumen. The Ingestible Micro‐Bio‐Electronic Device (IMBED) comprises a semi‐permeable membrane that separates the luminal space from an inner chamber holding genetically modified probiotic *E. coli* Nissle 1917.^100^ It has been described in proof‐of‐concept to detect heme, and has been expanded to detect other biomarkers, such as tetrathionate, which measures inflammation and acyl‐homoserine lactone, which is a signature of particular bacteria, which can be commensal or pathogenic.^100^ Further advances have led to a biosensing panel for nitric oxide, hydrogen peroxide, thiosulfate and tetrathionate, housed in a device that has been miniaturised to the extent that it can be safely orally ingested.^100^, ^101^

A novel sensing system that measures bacterial counts based on fluorometric signals, has been recently described in preliminary reports for small intestinal bacterial overgrowth.^102^, ^103^ Formal reporting of this study is awaited.

### 5.6 Pressure sensors

The SmartPill WMC houses two other sensors in addition to the pH sensor, including a single pressure sensor (measurement range 0 to 350 mmHg) (and the temperature sensor discussed below).^104^ This sensor has been used to assess gastrointestinal tract contractile frequency in healthy and gastroparetic subjects, although the clinical interpretation of these measures requires further consideration.^105^ One of the major difficulties interpreting pressure sensor data relates to the artefact generated from abdominal/body movement and transthoracic respiratory‐related movement. The inability to sub‐localise the device's location (e.g. antrum vs body vs fundus of stomach) is relevant, for example, in the evaluation of suspected gastric dysmotility, where the ability to differentially evaluate antral motility from fundus motility may be beneficial.

Pressure sensor use has also been evaluated in a pilot study of acute abdominal compartment syndrome in pigs using an ingestible microelectromechanical system (PressureDOT, 10 mm × 13 mm).^106^ Further work is required to assess its clinical applicability in humans.

### 5.7 Temperature sensors

Temperature sensors are common components of ingestible devices. Devices with a specific role in assessing core body temperature were first described in 1968 and remain commonly used in athletes or occupations involving thermally harsh environments.^107^ Examples include CorTemp Pill® (HQ Inc, USA) and its current version BodyCap—e‐Celsius performance pill (BodyCap, France), the VitalSense® capsule (Philips, USA) and the battery‐free, magnetically‐powered, myTemp (Nimegen, Netherlands).^108^ Other systems, such as the SmartPill WMC and Atmo Gas‐sensing capsule, use temperature sensors as adjunctive sensors, to confirm device ingestion (i.e. an increase in temperature relative to ambient temperature) and excretion (i.e. confirmed by a drop in temperature from body temperature to environmental).

## 6 INTERVENTIONAL FUNCTIONS OF INGESTIBLE DEVICES

Just as the clinical power of endoscopic examination of the gastrointestinal tract has been greatly enhanced by the ability to intervene with diagnostic and therapeutic actions, developing a device that has the capability of performing a ‘function’ during its transit through the gastrointestinal tract represents the next challenge for capsule technology. Various mechanisms may enable these functions to be performed, such as electrical, electrochemical, acoustic and thermal processes, leading, for example, to the opening of cavities for the targeted release of stored drugs or obtaining luminal samples. Multiple applications have been evaluated to date and examples are outlined below.

### 6.1 Assessment of drug adherence

Drug adherence is a significant issue in the management of chronic diseases, with rates as low as 30%–35% reported.^109^, ^110^ The first digital ingestion tracking system to monitor drug adherence was approved by the FDA in 2017 and consisted of a drug (aripiprazole) with an embedded ingestible sensor (Abilify MyCite, Otsuka Pharmaceutical Co. Ltd).^111^ This digital pill is activated in the stomach and interacts with a wearable ‘patch’, which communicates with a mobile application via Bluetooth® to confirm drug ingestion. Digital medication compliance has also been studied in other clinical settings in proof‐of‐concept and feasibility studies, including post‐renal transplant^112^ and treatment of tuberculosis.^113^

### 6.2 Drug delivery

In concept, ingestible devices offer a promising tool for targeted drug delivery to specific sites of the gastrointestinal tract. These may also provide a conduit for drugs that are considered otherwise unsuitable for oral delivery, such as peptides, which may be susceptible to enzymatic degradation, or likely to exhibit impaired absorption due to altered intestinal transit.^114^ Various mechanisms for drug delivery have been reported, with drug release under active or passive control. Passive release, for example, may be triggered by an external radiofrequency signal‐generated heat‐induced release of a spring, such as in the IntelliSite capsule (Scintipharma Inc., Lexington, KY, USA),^115^ while active release may occur, for example, via a motor‐controlled piston that can be activated manually using wireless data exchange or automatically, as seen in the IntelliCap capsule (Medimetrics Personalised Drug Delivery). The IntelliCap capsule contains a drug reservoir as well as pH and temperature sensors to help localise the capsule in the gastrointestinal tract in real‐time to allow targeted drug delivery.^116^, ^117^, ^118^ Such capsules can also be used for measuring gastrointestinal transit times and drug pharmacokinetics, and play a role in the assessment of regional drug absorption in clinical studies.^116^, ^117^, ^118^ Magnetic drug delivery devices, including robotic devices, have been designed but require ongoing refinement before they will be ready for clinical trials.^119^, ^120^

Various other models of drug delivery have been explored, including capsules with adhesion mechanisms to allow a sustained drug release, microneedle drug delivery technology, use of micromotors and the earlier mentioned ultrasound technology designed to increase mucosal permeability to drugs.^121^, ^122^, ^123^, ^124^, ^125^, ^126^, ^127^, ^128^ Electronic drug delivery devices have been reviewed in detail recently.^129^

### 6.3 Direct gastrointestinal tract sampling

Direct sampling of the gastrointestinal tract remains an important target in electronic device research and development, and would greatly enhance our understanding of gastrointestinal physiology and the microbiome in an unperturbed state. Most devices remain as aspirational projects and few, if any, are at the stage of commercialisation. The major challenges with these ingestible devices relate to obtaining a sufficient sample, that is leak‐proof, protected from contamination or alteration from gastrointestinal secretions and microbial metabolic activity and able to be accurately localised within the gastrointestinal tract to ensure that the site of sample is known, while remaining small enough to be safely ingested and delicate enough so as not to cause intestinal damage during the sampling process. The devices in the pipeline employ various techniques, such as spring‐loading, suction and osmosis, for example, to obtain gastrointestinal tract samples in one or more collecting channels and many remain electronic‐free.^130^, ^131^, ^132^ Tethered, non‐electronic capsules, such as the Krosby‐Kugler capsule, were used as early as the 1950s to collect small bowel samples.^133^, ^134^ A modified Intellicap system has recently been described and includes a collecting chamber for microbiome sampling and illustrates the expansive flexibility these technologies offer.^118^, ^135^ Various sampling devices have been described in prototype form, including a device able to collect small sample volumes in ex vivo and pig models, and a wireless device with magnetic actuation system, which is yet to be reported in vivo.^136^, ^137^, ^138^, ^139^, ^140^ Active control over timing and localisation of sampling remains under development, and advances in robotic technology will undoubtedly overcome many of these current limitations in the future.^141^, ^142^ At this stage, despite multiple reports describing novel designs with claims of proof‐of‐concept, none have provided key peer‐reviewed data that move the concepts into the realm of clinical reality. A logistical issue that is not likely to be overcome in the near future is the need to retrieve the excreted device in order to analyse the sampled material.

### 6.4 Vibration as the therapeutic intervention

Vibrating ingestible devices have been described recently for the management of constipation. ‘Vibrant’, a vibrating device marketed in the United States, received FDA approval for use in chronic idiopathic constipation in 2022 (Vibrant Ltd, Hakochav Yokneam, Israel). The battery‐operated device is encapsulated in a latex‐free plastic shell and contains a microchip and vibrating motor.^143^, ^144^, ^145^ It has been evaluated in a phase III randomised placebo‐controlled trial involving daily device ingestion (between 9 and 10 pm each day), 5 days a week for 8 weeks, with a pre‐programmed vibration schedule.^143^ Improvements in complete spontaneous bowel movements and stool characteristics were reported.^143^ Similar studies in China have been conducted using a vibrating electronic device (27 mm × 12 mm) which is activated via a smartphone (Vibravot, Ankon Medical Technology Co., Ltd).^146^ The 6‐h vibrating sequence is also pre‐programmed, designed to begin 8 h post‐ingestion but also has the ability to be externally controlled by a phone or configuration device.^146^

### 6.5 Other therapeutic interventions

The myriad of therapeutic interventional techniques capable of being performed by electronic devices seems only restricted by one's imagination. For example, robotic electroceutical capsules that, by delivering controlled electrical stimulation to the enteric nervous system, might influence local motility, such as gastric emptying,^147^ or the gut‐brain axis, such as by modulating hunger‐related hormones^148^ and intra‐gastric balloon capsules for obesity management^149^ are under development or have been proposed. Robotic control of gastrointestinal haemorrhage has been proposed based on the detection of bleeding detected by blood‐coloured pixels with subsequent inflation of a balloon from an endothermic reaction to apply pressure to the site of haemorrhage,^150^ or by magnetically steering a device with a surgical clip in situ, which is able to be deployed at the site of bleeding.^151^ Origami robots that unfold from a swallowed device and are subsequently controlled by external magnetic fields can be directed to perform a therapeutic function, like removing a swallowed button battery or patching a wound, which have also been proposed.^152^

## 7 INGESTIBLE ELECTRONIC DEVICES IN CLINICAL PRACTICE

**FIGURE 2:**
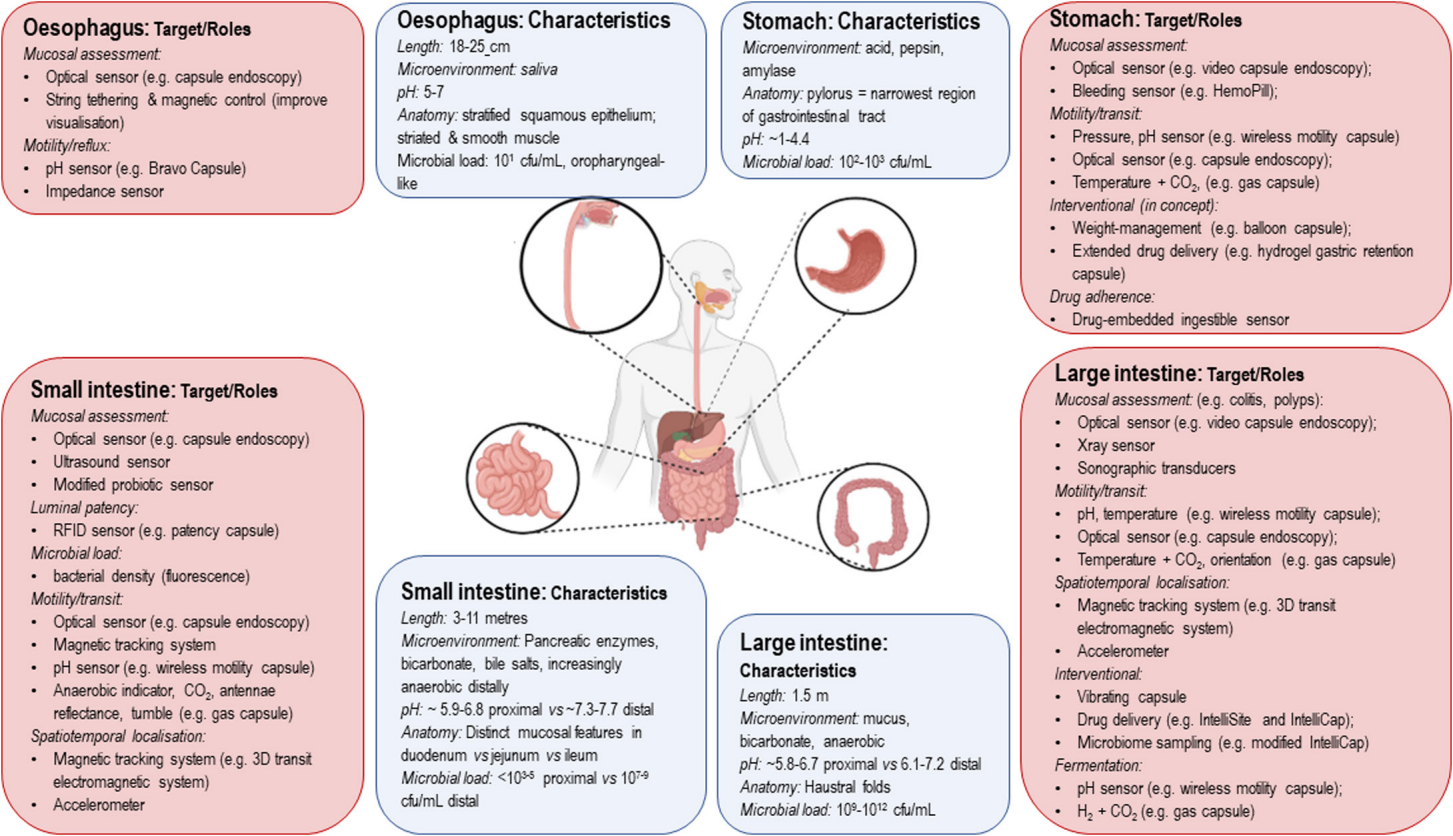
Illustrates the typical characteristics of each section of the gastrointestinal tract from oesophagus to large intestine (light blue) and many potential targets for ingestible electronic device technology (peach). cfu, colony forming unit; CO_2_, carbon dioxide; H_2_, hydrogen gas.

The roles, potential or current, of ingestible electronic devices are summarised according to gastrointestinal regions in Figure 2. There are several important considerations required during the development of a device intended for clinical use, which are outlined in Table 1. Beyond the obvious technical issues of developing the device itself and ensuring it is safe, reliable and affordable, the incorporation of these technologies into clinical practice requires multidisciplinary collaboration. This is to ensure the device is measuring something that is of clinical relevance, be it for understanding an individual's physiology or a specific pathophysiological process and, ultimately, is what a clinician needs and wants when treating their patients. Validating the information captured by this technology to confirm it is real and accurate is challenging when there may be no precedent information available for comparison. A second challenge relates to incorporating this new biological or physiological information into established clinical algorithms. Thirdly, the demonstration of cost‐effectiveness so that this innovative technology and information can be translated into clinical funding models is essential in the current tight economic climate. In the earlier stages of research and development of these devices, funding is even more challenging, due to the high costs of running development programs and the extensive investigations required to prove efficacy and commercial viability and, ultimately, administrative approval be it FDA or TGA. However, over the longer term, ingestible electronic devices have the potential to be a cost‐effective tool due to the lack of stationary equipment required to gather the data in a patient who often can remain ambulatory and in an outpatient setting. In fact, these technologies have the potential to provide far‐reaching access to healthcare tests that would otherwise be inaccessible without dedicated travel to a hospital or medical centre.

**TABLE 1:** Advantages of ingestible electronic devices in clinical practice and the challenges that must be addressed.

*Advantages*	
Safety	The implications for this are that they can potentially be ingested on repeat occasions (e.g. to monitor response to treatment)
Accessibility	Enhanced for patients who may not otherwise have the finances or proximity to centres that offer conventional testing
Convenience	Able to be administered in the consultation office avoiding otherwise cumbersome techniques/medical investigations
Unmodified physiological assessment	Data can be collected in a manner that allows the subject to continue their activities of daily living largely unchanged, with minimal disruption and without the need for bowel preparation or sedation, which may be required with other techniques such as colonoscopy
Longitudinal data collection	Unlike conventional methods such as breath test or biopsy, ingestible capsules offer the ability to provide longitudinal data often spanning multiple anatomical segments, providing dynamic insight into the physiology and anatomy of the gastrointestinal tract
Efficient transfer of data	The ability for real‐time data transmission enables real‐time assessment by the physician, including while the device is in transit, providing the opportunity to alter the capsule's operating characteristics such as to improve its completion rate or to feedback results to a patient
Environmental footprint	Capsule endoscopy may reduce the carbon footprint by reducing the need for conventional endoscopy.^153^
*Challenges*	
Device‐associated issues	Technical failure reports vary widely (e.g. 0.8%–10% for WMC; up to 9% for capsule endoscopy).^82^, ^154^, ^155^ Test completion rates may be enhanced by maximising device data transmission distance (i.e. choice of antennae, battery, transmission frequency and optimal BMI or central adiposity ranges), safeguarding against/limiting interference from environmental structures (e.g. steel beds, mobile phones/other electronic devices), and simplifying the testing protocol
Device safety	ISO‐10993 International Standard for Biological Evaluation of Medical Devices provides a framework and standard against which devices must be compared.; IEC‐60601 for safety and performance of the electronic system; ISO‐62304 for Software
Data integrity	Data collected must not be manipulated or contaminated (e.g. interference from other devices)
Data validation and interpretation	The unique measurements obtained by capsules are plagued by the lack of ‘gold‐standard’ techniques by which to compare the data. This creates challenges with data interpretation and its subsequent translation into clinical practice
External control over the device	The capacity to provide active control over the device and fine‐tuned locomotion is challenging due to the various dimensions of the gastrointestinal tract (e.g. capacious stomach, narrow small bowel lumen) and variable, unpredictable rates of gastrointestinal peristalsis
Capsule localisation	Current devices are limited to broad gastrointestinal regional localisation (e.g. gastric, small intestine etc.). Further refinements in capsule localisation will enable more detailed mapping of the gastrointestinal tract according to form and function
Incorporation of interventional modes	One of the limitations of capsule endoscopy is its inability to provide therapeutic intervention once an abnormality is detected. The ability to incorporate an interventional mode to these capsules remains an important goal. Multiple challenges still exist with the refinement of robotic technology, including miniaturisation, power, localisation and safety (perforation, gastrointestinal wall strain, gastrointestinal tract retention).^150^
Data safety and ownership	Secure storage of data (e.g. in the cloud) is essential in the healthcare setting.^156^ Ownership of the data and its storage has legal and ethical ramifications, which extend beyond the patient and physician and also include the third party who markets the device.^157^
Costs	Initial development costs of electronic ingestible devices are high, but the cost of production and maintenance are likely to fall over time, and generally cost less than standard analytical techniques (e.g. gas chromatography).^158^ Health economic benefits may be seen in the future as these devices provide the opportunity for preventive health strategies such as screening and/or early intervention
Achieving reimbursement from the ‘payers’	Convincing the ‘payers’ that there is an economic reason to change from the traditional paradigm and/or that the information or functions provided by the device have sufficient clinical value to warrant a possibly higher cost. Clinicians may be willing to use them, but, if the payers are not convinced, they will not gain traction
Patient‐related considerations	Minimally‐intrusive testing protocol, such that the patient is not inconvenienced by a cumbersome data receiver and/or required to significantly adjust their usual daily activities (e.g. avoid high‐intensity activity) for extended periods of time. Development of simplified testing equipment to minimise the opportunity for patient error (e.g. wearable device vs carried device)
Environmental footprint	Considerations should be made for environmentally friendly disposal pathways

The story of capsule endoscopy has demonstrated that new technologies can be successfully incorporated into clinical practice and funding models. Its clinical acceptability and application were facilitated by the fact that endoscopic imaging of gastrointestinal mucosa was already standard practice. Hence, the introduction of a low‐risk method of visualising the entire small intestine was eagerly endorsed by the gastroenterological community. By contrast, the introduction of a validated, low‐risk and convenient method of measuring regional transit times (i.e. the SmartPill) was limited. This may have been related to the uncertainty of the impact of the unique data obtained on clinical decision‐making despite the demonstration of its value^159^ and inability in many environments to gain funding for its use due to uncertain cost–benefit (i.e. it was perceived as unaffordable). As ingestible device technology expands, more examples of this will arise and will be associated with these clinical challenges. The recent novel demonstration that the locoregional colonic distribution of carbohydrate fermentation,^74^ and thus short‐chain fatty acid delivery, can be evaluated using hydrogen concentrations is another example. Colonic fermentation and where it is occurring in the colon are not mainstream concerns for clinicians because such knowledge has had no prior clinical correlate or measurement until now and so it is likely that the clinical impact and awareness of this new information will take some time to be established.

### 7.1 Safety practice points for consideration with ingestible devices

In general, the safety profiles of the ingestible devices currently commercially available are acceptable with infrequent reporting of serious complications. Ultimately, the nature and magnitude of the risks are dependent on the type of device being used and its indication. The clinician should take into consideration these risks, as outlined in Table 2. Clearly, some of these clinical scenarios have little to no data upon which the risk can be quantified, but the balance of risk and benefit still has to be evaluated in line with good clinical practice.

**TABLE 2:** Risks and considerations for the application of telemetric capsules in clinical gastroenterological practice.^154^, ^160^

Known or suspected structuring or stenosing disease of the bowel (including inflammatory bowel disease^a^, oesophageal strictures, pyloric stenosis)
Known or suspected gastrointestinal tract fistulae
Prior history of intestinal surgery (bowel resection), complex or recent intra‐abdominal surgery (<3 months)
Known adhesions
History of bowel obstruction
Prior history of excessive or prolonged NSAID use (ESGE guidelines recommend at least 4 week cessation^33^)
Diverticular disease (including oesophageal or severe colonic diverticular disease) or current diverticulitis
Prior abdominal radiotherapy
Swallowing disorders, including achalasia or oropharyngeal dysfunction
Prior gastric bezoar (which may trap the capsule)
Pregnancy (due to the lack of safety data)
Non‐compliance (including Dementia or intellectual impairment)
Implantable/Cardiac devices^b^

^a^ See text regarding Crohn's disease.

^b^ ESGE guidelines suggest safety to proceed with capsule endoscopy in pacemaker and implantable device albeit low‐quality evidence. Best refer to specific manufacturer statement.

One particular adverse event is capsule retention, defined as retention of the device within the gastrointestinal tract for 2 weeks or more.^161^ Small devices (e.g. less than 11 mm × 26 mm) of low density have minimal risk of retention in a healthy individual and can pass through the narrowest region of the gastrointestinal tract, the pylorus. Reported retention rates vary according to the specific device and its indication for use, ranging from 0.3% for WMC transit assessment and about 2% up to 8.2% in capsule endoscopy for suspected small bowel bleeding and established inflammatory bowel disease, respectively.^154^, ^162^ Patency capsules, such as the Agile Patency Capsule, as discussed earlier, may be used to assess the risk of retention (measuring 26 mm × 11 mm).^62^ Whether the patency capsule used needs to be an exact replica of the intended test device in terms of size and density, or whether a similar device can be used, is uncertain. Capsule retention management may be simply observational in nature or require endoscopic or surgical retrieval, which will be determined by the location of the retained capsule and whether the patient is symptomatic.^161^

Problems associated with swallowing devices have been reported uncommonly. For example, post‐marketing studies report a rate of 0.6% for swallowing difficulties with the WMC and 1.5% for capsule endoscopy.^154^ Aspiration has occurred at a rate of approximately 1:1000 in capsule endoscopy studies, which is more common in older patients with reported dysphagia.^163^, ^164^ Where dysphagia is an issue or the device is too large to be safely ingested, endoscopic insertion using a capsule‐loading device is available to deliver the capsule to the stomach or duodenum.

## 8 CONCLUSION: THE FUTURE OF INGESTIBLE ELECTRONIC DEVICES IN GASTROENTEROLOGY

That ingestible devices have a place in gastroenterological practice is not in question—capsule endoscopy is viable and technology‐driven, and, in many ways, has revolutionised imaging of the small bowel. Newer technological advances will undoubtedly continue to expand the role of ingestible electronic device technology in gastroenterology. Enhanced optics and sensing will increase diagnostic capabilities and indications for use, and it is highly likely that refinements and additions to the list of interventional capabilities of the capsule means that its role in therapy will also increase in the future, with multimodal devices likely to be developed, ultimately under robotic control. Beyond the scope of this review but worthy of mention is the role artificial intelligence will play in this sphere, particularly with regard to machine learning and computer‐aided diagnoses.

While still in relative infancy, the role of ingestible devices in gastroenterology is exciting and provides an opportunity to greatly expand our understanding of gastrointestinal physiology and indeed pathophysiology in a relatively undisturbed and minimally invasive way. It also provides a tool by which changes that may occur in response to pharmacological, dietary or other health interventions over time can be monitored. Studies programmed for each individual's unique clinical circumstances, with assessment and delivery of treatments or substrates promise to herald a new era of personalised medicine.

## AUTHOR CONTRIBUTIONS

**Phoebe A. Thwaites:** Conceptualization; writing – original draft; writing – review and editing. **Chu K. Yao:** Writing – review and editing. **Emma P. Halmos:** Writing – review and editing. **Jane G. Muir:** Writing – review and editing. **Rebecca E. Burgell:** Writing – review and editing. **Kyle J. Berean:** Writing – review and editing. **Kourosh Kalantar‐zadeh:** Conceptualization; writing – review and editing. **Peter R. Gibson:** Conceptualization; writing – review and editing.

## FUNDING INFORMATION

PAT is a recipient of an NHMRC postgraduate scholarship.

## CONFLICT OF INTEREST STATEMENT

Phoebe A. Thwaites: None to declare. Chu K. Yao: Recipient of Atmo Biosciences research support. Emma P. Halmos: Received research grants for investigator‐driven studies from Mindset Health, and speaker honoraria from Sandoz Pty Ltd and Mindset Health. Jane G. Muir: Her department financially benefits from the sale of digital application and booklets on the FODMAP diet. Rebecca E. Burgell: Consultant or advisory board member for Allergan, Atmo Biosciences, Antara. She has received speaking honoraria from Bayer. Kyle J. Berean: Employee and shareholder of Atmo Biosciences. Kourosh Kalantar‐zadeh: Has consulted for Atmo Biosciences. Peter R. Gibson: Consultant or advisory board member for Anatara, Atmo Biosciences, Immunic Therapeutics, Novoviah, Intrinsic Medicine, Topas and Comvita. He has received research grants for investigator‐driven studies from Atmo Biosciences and Mindset Health, and speaker honoraria from Dr Falk Pharma and Mindset Health. He holds shares in Atmo Biosciences. His department financially benefits from the sale of digital application and booklets on the FODMAP diet.

## References

[1] Kalantar‐zadeh K , Ha N , Ou JZ , Berean KJ . Ingestible sensors. ACS Sens. 2017;2(4):468–483. 28723186 10.1021/acssensors.7b00045

[2] Alazemi AJ , Iqbal A . A compact and wideband MIMO antenna for high‐data‐rate biomedical ingestible capsules. Sci Rep. 2022;12(1):14290. 35995821 10.1038/s41598-022-18468-2 PMC9395510

[3] Sauer DU . SECONDARY BATTERIES – LEAD – ACID SYSTEMS | lifetime determining processes. In: Garche J , editor. Encyclopedia of electrochemical power sources. Amsterdam: Elsevier; 2009. p. 805–815.

[4] Swain P . At a watershed? Technical developments in wireless capsule endoscopy. J Dig Dis. 2010;11(5):259–265. 20883421 10.1111/j.1751-2980.2010.00448.x

[5] Sharifi M , Pothu R , Boddula R , Bardajee GR . Trends of biofuel cells for smart biomedical devices. Int J Hydrogen Energy. 2021;46(4):3220–3229.

[6] de la Paz E , Maganti NH , Trifonov A , Jeerapan I , Mahato K , Yin L , et al. A self‐powered ingestible wireless biosensing system for real‐time in situ monitoring of gastrointestinal tract metabolites. Nat Commun. 2022;13(1):7405. 36456568 10.1038/s41467-022-35074-y PMC9715945

[7] Christoe MJ , Han J , Kalantar‐Zadeh K . Telecommunications and data processing in flexible electronic systems. Adv Mater Technol. 2020;5(1):1900733.

[8] Biswas B , Karmakar A , Chandra V . Miniaturised wideband ingestible antenna for wireless capsule endoscopy. IET Microw Antennas Propag. 2020;14(4):293–301.

[9] Christoe MJ , Yuan J , Michael A , Kalantar‐Zadeh K . Bluetooth signal attenuation analysis in human body tissue analogues. IEEE Access. 2021;9:85144–85150.

[10] Waimin JF , Nejati S , Jiang H , Qiu J , Wang J , Verma MS , et al. Smart capsule for non‐invasive sampling and studying of the gastrointestinal microbiome. RSC Adv. 2020;10(28):16313–16322. 35498852 10.1039/c9ra10986b PMC9052936

[11] Enns C , Galorport C , Ou G , Enns R . Assessment of capsule endoscopy utilizing Capsocam plus in patients with suspected small bowel disease including pilot study with remote access patients during pandemic. J Can Assoc Gastroenterol. 2021;4(6):269–273. 34988365 10.1093/jcag/gwaa042 PMC8697548

[12] Hanscom M , Cave DR . Endoscopic capsule robot‐based diagnosis, navigation and localization in the gastrointestinal tract. Front Robot AI. 2022;9:1–16. 10.3389/frobt.2022.896028 PMC9479458 36119725

[13] Glass P , Cheung E , Sitti M . A legged anchoring mechanism for capsule endoscopes using micropatterned adhesives. IEEE Trans Biomed Eng. 2008;55(12):2759–2767. 19126455 10.1109/TBME.2008.2002111

[14] Valdastri P , Webster RJ , Quaglia C , Quirini M , Menciassi A , Dario P . A new mechanism for mesoscale legged locomotion in compliant tubular environments. IEEE Trans Robot. 2009;25(5):1047–1057.

[15] Kim HM , Yang S , Kim J , Park S , Cho JH , Park JY , et al. Active locomotion of a paddling‐based capsule endoscope in an in vitro and in vivo experiment (with videos). Gastrointest Endosc. 2010;72(2):381–387. 20497903 10.1016/j.gie.2009.12.058

[16] Falco ID , Tortora G , Dario P , Menciassi A . An integrated system for wireless capsule endoscopy in a liquid‐distended stomach. IEEE Trans Biomed Eng. 2014;61(3):794–804. 24216631 10.1109/TBME.2013.2290018

[17] Liao Z , Hou X , Lin‐Hu E‐Q , Sheng JQ , Ge ZZ , Jiang B , et al. Accuracy of magnetically controlled capsule endoscopy, compared with conventional gastroscopy, in detection of gastric diseases. Clin Gastroenterol Hepatol. 2016;14(9):1266–1273.e1261. 27211503 10.1016/j.cgh.2016.05.013

[18] Jiang B , Pan J , Qian YY , He C , Xia J , He SX , et al. Clinical guideline on magnetically controlled capsule gastroscopy (2021 edition). J Dig Dis. 2023;24(2):70–84. 37220999 10.1111/1751-2980.13173

[19] Pandolfino JE , Richter JE , Ours T , Guardino JM , Chapman J , Kahrilas PJ . Ambulatory esophageal pH monitoring using a wireless system. Am J Gastroenterol. 2003;98(4):740–749. 12738450 10.1111/j.1572-0241.2003.07398.x

[20] Ou JZ , Cottrell JJ , Ha N , Pillai N , Yao CK , Berean KJ , et al. Potential of in vivo real‐time gastric gas profiling: a pilot evaluation of heat‐stress and modulating dietary cinnamon effect in an animal model. Sci Rep. 2016;6:33387. 27633400 10.1038/srep33387 PMC5025890

[21] Mau MM , Sarker S , Terry BS . Ingestible devices for long‐term gastrointestinal residency: a review. Progr Biomed Eng. 2021;3(4):1–18.

[22] Iddan G , Meron G , Glukhovsky A , Swain P . Wireless capsule endoscopy. Nature. 2000;405(6785):417. 10.1038/35013140 10839527

[23] Appleyard M , Fireman Z , Glukhovsky A , Jacob H , Shreiver R , Kadirkamanathan S , et al. A randomized trial comparing wireless capsule endoscopy with push enteroscopy for the detection of small‐bowel lesions. Gastroenterology. 2000;119(6):1431–1438. 11113063 10.1053/gast.2000.20844

[24] Adler SN , Metzger YC . PillCam COLON capsule endoscopy: recent advances and new insights. Therap Adv Gastroenterol. 2011;4(4):265–268. 10.1177/1756283X11401645 PMC3131168 21765870

[25] Eliakim R , Fireman Z , Gralnek IM , Yassin K , Waterman M , Kopelman Y , et al. Evaluation of the PillCam colon capsule in the detection of colonic pathology: results of the first multicenter, prospective, comparative study. Endoscopy. 2006;38(10):963–970. 17058158 10.1055/s-2006-944832

[26] Cave DR , Fleischer DE , Leighton JA , Faigel DO , Heigh RI , Sharma VK , et al. A multicenter randomized comparison of the endocapsule and the Pillcam SB. Gastrointest Endosc. 2008;68(3):487–494. 18410941 10.1016/j.gie.2007.12.037

[27] Bang S , Park JY , Jeong S , Kim YH , Shim HB , Kim TS , et al. First clinical trial of the “MiRo” capsule endoscope by using a novel transmission technology: electric‐field propagation. Gastrointest Endosc. 2009;69(2):253–259. 18640676 10.1016/j.gie.2008.04.033

[28] Friedrich K , Gehrke S , Stremmel W , Sieg A . First clinical trial of a newly developed capsule endoscope with panoramic side view for small bowel: a pilot study. J Gastroenterol Hepatol. 2013;28(9):1496–1501. 23701674 10.1111/jgh.12280

[29] Liao Z , Gao R , Li F , Xu C , Zhou Y , Wang JS , et al. Fields of applications, diagnostic yields and findings of OMOM capsule endoscopy in 2400 Chinese patients. World J Gastroenterol. 2010;16(21):2669–2676. 20518090 10.3748/wjg.v16.i21.2669 PMC2880781

[30] Mehedi IM , Rao KP , Alotaibi FM , Alkanfery HM . Intelligent wireless capsule endoscopy for the diagnosis of gastrointestinal diseases. Diagnostics (Basel). 2023;13(8):1–23. 10.3390/diagnostics13081445 PMC10137352 37189546

[31] Li Z , Carter D , Eliakim R , Zou W , Wu H , Liao Z , et al. The current main types of capsule endoscopy. In: Li Z , Liao Z , McAlindon M , editors. Handbook of capsule endoscopy. Dordrecht: Springer Netherlands; 2014. p. 5–45.

[32] Pennazio M , Rondonotti E , Despott EJ , Dray X , Keuchel M , Moreels T , et al. Small‐bowel capsule endoscopy and device‐assisted enteroscopy for diagnosis and treatment of small‐bowel disorders: European Society of Gastrointestinal Endoscopy (ESGE) guideline – update 2022. Endoscopy. 2022;55(1):58–95. 36423618 10.1055/a-1973-3796

[33] Pennazio M , Spada C , Eliakim R , Keuchel M , May A , Mulder C , et al. Small‐bowel capsule endoscopy and device‐assisted enteroscopy for diagnosis and treatment of small‐bowel disorders: European Society of Gastrointestinal Endoscopy (ESGE) clinical guideline. Endoscopy. 2015;47(4):352–386. 25826168 10.1055/s-0034-1391855

[34] Ahmed M . Video capsule endoscopy in gastroenterology. Gastroenterology Res. 2022;15(2):47–55. 35572472 10.14740/gr1487 PMC9076159

[35] Naymagon S , Naymagon L , Wong SY , Ko HM , Renteria A , Levine J , et al. Acute graft‐versus‐host disease of the gut: considerations for the gastroenterologist. Nat Rev Gastroenterol Hepatol. 2017;14(12):711–726. 28951581 10.1038/nrgastro.2017.126 PMC6240460

[36] Enns RA , Hookey L , Armstrong D , Bernstein CN , Heitman SJ , Teshima C , et al. Clinical practice guidelines for the use of video capsule endoscopy. Gastroenterology. 2017;152(3):497–514. 28063287 10.1053/j.gastro.2016.12.032

[37] Malagelada C , de Iorio F , Azpiroz F , Accarino A , Segui S , Radeva P , et al. New insight into intestinal motor function via noninvasive endoluminal image analysis. Gastroenterology. 2008;135(4):1155–1162. 18691579 10.1053/j.gastro.2008.06.084

[38] Kim HM , Choi JS , Cho JH . A pilot trial of ambulatory monitoring of gastric motility using a modified magnetic capsule endoscope. J Neurogastroenterol Motil. 2014;20(2):261–264. 24840379 10.5056/jnm.2014.20.2.261 PMC4015206

[39] Singh A , Marshall C , Chaudhuri B , Okoli C , Foley A , Person SD , et al. Timing of video capsule endoscopy relative to overt obscure GI bleeding: implications from a retrospective study. Gastrointest Endosc. 2013;77(5):761–766. 23375526 10.1016/j.gie.2012.11.041

[40] Carpi F , Galbiati S , Carpi A . Magnetic shells for gastrointestinal endoscopic capsules as a means to control their motion. Biomed Pharmacother. 2006;60(8):370–374. 16935464 10.1016/j.biopha.2006.07.001

[41] Xiao Y‐F , Wu Z‐X , He S , Zhou YY , Zhao YB , He JL , et al. Fully automated magnetically controlled capsule endoscopy for examination of the stomach and small bowel: a prospective, feasibility, two‐centre study. Lancet Gastroenterol Hepatol. 2021;6(11):914–921. 34555347 10.1016/S2468-1253(21)00274-0

[42] Keller J , Fibbe C , Volke F , Gerber J , Mosse AC , Reimann‐Zawadzki M , et al. Inspection of the human stomach using remote‐controlled capsule endoscopy: a feasibility study in healthy volunteers (with videos). Gastrointest Endosc. 2011;73(1):22–28. 21067740 10.1016/j.gie.2010.08.053

[43] Keller J , Fibbe C , Volke F , Gerber J , Mosse AC , Reimann‐Zawadzki M , et al. Remote magnetic control of a wireless capsule endoscope in the esophagus is safe and feasible: results of a randomized, clinical trial in healthy volunteers. Gastrointest Endosc. 2010;72(5):941–946. 20855064 10.1016/j.gie.2010.06.053

[44] Liao Z , Zou W , Li Z‐S . Clinical application of magnetically controlled capsule gastroscopy in gastric disease diagnosis: recent advances. Sci China Life Sci. 2018;61(11):1304–1309. 30367341 10.1007/s11427-018-9353-5

[45] Jiang B , Qian Y‐Y , Wang Y‐C , Pan J , Jiang X , Zhu JH , et al. A novel capsule endoscopy for upper and mid‐GI tract: the UMGI capsule. BMC Gastroenterol. 2023;23(1):76. 36927462 10.1186/s12876-023-02696-5 PMC10019395

[46] Wang S , Huang Y , Hu W , Mao H , McAlindon ME , Liu Y , et al. Detachable string magnetically controlled capsule endoscopy for detecting high‐risk varices in compensated advanced chronic liver disease (CHESS1801): a prospective multicenter study. Lancet Reg Health West Pac. 2021;6:100072. 34327406 10.1016/j.lanwpc.2020.100072 PMC8315440

[47] Zhang Y , Zhang Y , Huang X . Development and application of magnetically controlled capsule endoscopy in detecting gastric lesions. Gastroenterol Res Pract. 2021;2021:2716559. 35003252 10.1155/2021/2716559 PMC8739542

[48] Zhao A‐J , Qian Y‐Y , Sun H , Hou X , Pan J , Liu X , et al. Screening for gastric cancer with magnetically controlled capsule gastroscopy in asymptomatic individuals. Gastrointest Endosc. 2018;88(3):466–474.e461. 29753039 10.1016/j.gie.2018.05.003

[49] Mahmood S , Schostek S , Schurr MO , Bergsland J , Balasingham I , Fosse E . Robot‐assisted magnetic capsule endoscopy;navigating colorectal inclinations. Minim Invasive Ther Allied Technol. 2022;31(6):930–938. 35112641 10.1080/13645706.2022.2032181

[50] Schostek S , Schurr MO . The HemoCop Telemetric Sensor System: technology and results of in‐vivo assessment. Stud Health Technol Inform. 2012;177:97–100. 22942037

[51] Schostek S , Zimmermann M , Keller J , Fode M , Melbert M , Schurr MO , et al. Telemetric real‐time sensor for the detection of acute upper gastrointestinal bleeding. Biosens Bioelectron. 2016;78:524–529. 26667093 10.1016/j.bios.2015.11.073

[52] Nakamura K . Further development of endoscopic imaging: “era of light” activities with optics and image processing technology. In: Niwa H , Tajiri H , Nakajima M , Yasuda K , editors. New Challenges in Gastrointestinal Endoscopy. Tokyo: Springer.

[53] Anbarasan T , Démoré CEM , Lay H , Sunoqrot MR , Poltarjonoks R , Cochran S , et al. High resolution microultrasound (μUS) investigation of the gastrointestinal (GI) tract. Methods Mol Biol. 2017;1572:541–561. 28299710 10.1007/978-1-4939-6911-1_34

[54] Cox BF , Stewart F , Lay H , Cummins GC , Newton IP , Desmulliez MPY , et al. Ultrasound capsule endoscopy: sounding out the future. Ann Transl Med. 2017;5(9):201. 28567381 10.21037/atm.2017.04.21 PMC5438792

[55] Wiersema MJ , Wiersema LM . High‐resolution 25‐megahertz ultrasonography of the gastrointestinal wall: histologic correlates. Gastrointest Endosc. 1993;39(4):499–504. 8365596 10.1016/s0016-5107(93)70159-5

[56] Lay HS , Qiu Y , Al‐Rawhani M , Beeley J , Poltarjonoks R , Seetohul V , et al. Progress towards a multi‐modal capsule endoscopy device featuring microultrasound imaging. Paper presented at: 2016 IEEE International Ultrasonics Symposium (IUS); 2016. Tours, France. p. 1–4.

[57] Cummins G , Desmulliez MPY , Watson DEG , Mitrakos V , Faerber J , Demore CEM , et al. Sonopill: a platform for gastrointestinal disease diagnosis and therapeutics. Paper presented at 6th Joint Workshop on New Technologies for Computer/Robot Assisted Surgery. 2016, Pisa, Italy.

[58] Stewart F , Cummins G , Turcanu MV , Cox BF , Prescott A , Clutton E , et al. Ultrasound mediated delivery of quantum dots from a proof of concept capsule endoscope to the gastrointestinal wall. Sci Rep. 2021;11(1):2584. 33510366 10.1038/s41598-021-82240-1 PMC7844260

[59] Qiu Y , Huang Y , Zhang Z , Cox BF , Liu R , Hong J , et al. Ultrasound capsule endoscopy with a mechanically scanning micro‐ultrasound: a porcine study. Ultrasound Med Biol. 2020;46(3):796–804. 31902446 10.1016/j.ultrasmedbio.2019.12.003

[60] Norton JC , Slawinski PR , Lay HS , Martin JW , Cox BF , Cummins G , et al. Intelligent magnetic manipulation for gastrointestinal ultrasound. Sci Robot. 2019;4(31):eaav7725. 31380501 10.1126/scirobotics.aav7725 PMC6677276

[61] Gluck N , Shpak B , Brun R , Rösch T , Arber N , Moshkowitz M . A novel prepless X‐ray imaging capsule for colon cancer screening. Gut. 2016;65(3):371–373. 26628510 10.1136/gutjnl-2015-310893 PMC4789826

[62] Caunedo‐Alvarez A , Romero‐Vazquez J , Herrerias‐Gutierrez JM . Patency and agile capsules. World J Gastroenterol. 2008;14(34):5269–5273. 18785278 10.3748/wjg.14.5269 PMC2744056

[63] Sharma S , Ramadi KB , Poole NH , Srinivasan SS , Ishida K , Kuosmanen J , et al. Location‐aware ingestible microdevices for wireless monitoring of gastrointestinal dynamics. Nat Electron. 2023;6(3):242–256. 37745833 10.1038/s41928-023-00916-0 PMC10516531

[64] Worsoe J , Fynne L , Gregersen T , Schlageter V , Christensen LA , Dahlerup JF , et al. Gastric transit and small intestinal transit time and motility assessed by a magnet tracking system. BMC Gastroenterol. 2011;11:145. 22206545 10.1186/1471-230X-11-145 PMC3295650

[65] Stathopoulos E , Schlageter V , Meyrat B , Ribaupierre Y , Kucera P . Magnetic pill tracking: a novel non‐invasive tool for investigation of human digestive motility. Neurogastroenterol Motil. 2005;17(1):148–154. 15670274 10.1111/j.1365-2982.2004.00587.x

[66] Hiroz P , Schlageter V , Givel J‐c , Kucera P . Colonic movements in healthy subjects as monitored by a magnet tracking system. Neurogastroenterol Motil. 2009;21(8):838–e857. 19400925 10.1111/j.1365-2982.2009.01298.x

[67] Poulsen JL , Nilsson M , Brock C , Sandberg TH , Krogh K , Drewes AM . The impact of opioid treatment on regional gastrointestinal transit. J Neurogastroenterol Motil. 2016;22(2):282–291. 26811503 10.5056/jnm15175 PMC4819867

[68] Gregersen T , Haase A‐M , Schlageter V , Gronbaek H , Krogh K . Regional gastrointestinal transit times in patients with carcinoid diarrhea: assessment with the novel 3D‐transit system. J Neurogastroenterol Motil. 2015;21(3):423–432. 26130638 10.5056/jnm15035 PMC4496908

[69] Haase AM , Gregersen T , Schlageter V , Scott MS , Demierre M , Kucera P , et al. Pilot study trialling a new ambulatory method for the clinical assessment of regional gastrointestinal transit using multiple electromagnetic capsules. Neurogastroenterol Motil. 2014;26(12):1783–1791. 25348504 10.1111/nmo.12461

[70] Mark EB , Poulsen JL , Haase AM , Frøkjaer JB , Schlageter V , Scott SM , et al. Assessment of colorectal length using the electromagnetic capsule tracking system: a comparative validation study in healthy subjects. Colorectal Dis. 2017;19(9):O350–O357. 28688203 10.1111/codi.13810

[71] Nandhra GK , Mark EB , di Tanna GL , Haase AM , Poulsen J , Christodoulides S , et al. Normative values for region‐specific colonic and gastrointestinal transit times in 111 healthy volunteers using the 3D‐transit electromagnet tracking system: influence of age, gender, and body mass index. Neurogastroenterol Motil. 2020;32(2):e13734. 31565841 10.1111/nmo.13734

[72] Evenson KR , Goto MM , Furberg RD . Systematic review of the validity and reliability of consumer‐wearable activity trackers. Int J Behav Nutr Phys Act. 2015;12(1):159. 26684758 10.1186/s12966-015-0314-1 PMC4683756

[73] Raj R , Ussavarungsi K , Nugent K . Accelerometer‐based devices can be used to monitor sedation/agitation in the intensive care unit. J Crit Care. 2014;29(5):748–752. 24973100 10.1016/j.jcrc.2014.05.014

[74] So D , Yao C , Gill P , Thwaites P , Ardalan Z , McSweeney C , et al. Detection of changes in regional colonic fermentation in response to supplementing a low FODMAP diet with dietary fibres by hydrogen concentration, but not by luminal pH. Aliment Pharmacol Ther. 2023;58:417–428. 37386938 10.1111/apt.17629 PMC10946934

[75] Thwaites PA , Yao CK , Maggo J , John J , Chrimes AF , Burgell RE , et al. Comparison of gastrointestinal landmarks using the gas‐sensing capsule and wireless motility capsule. Aliment Pharmacol Ther. 2022;56(9):1337–1348. 36082475 10.1111/apt.17216 PMC9826325

[76] Yarbrough DR , McAlhany JC , Cooper N , Weidner MG . Evaluation of the Heidelberg pH capsule: method of tubeless gastric analysis. Am J Surg. 1969;117(2):185–192. 5773932 10.1016/0002-9610(69)90303-1

[77] Branicki FJ , Evans DF , Ogilvie AL , Atkinson M , Hardcastle JD . Ambulatory monitoring of oesophageal pH in reflux oesophagitis using a portable radiotelemetry system. Gut. 1982;23(11):992–998. 7129208 10.1136/gut.23.11.992 PMC1419793

[78] Tharavej C , Hagen JA , Portale G , Hsieh CC , Gandamihardja TAK , Lipham JC , et al. Bravo capsule induction of esophageal hypercontractility and chest pain. Surg Endosc Other Interv Tech. 2006;20(5):783–786. 10.1007/s00464-005-0257-8 16544080

[79] Wong W‐M , Bautista J , Dekel R , Malagon IB , Tuchinsky I , Green C , et al. Feasibility and tolerability of transnasal/per‐oral placement of the wireless pH capsule vs. traditional 24‐h oesophageal pH monitoring – a randomized trial. Aliment Pharmacol Ther. 2005;21(2):155–163. 15679765 10.1111/j.1365-2036.2005.02313.x

[80] Gonzalez‐Guillaumin JL , Sadowski DC , Kaler KVIS , Mintchev MP . Ingestible capsule for impedance and pH monitoring in the esophagus. IEEE Trans Biomed Eng. 2007;54(12):2231–2236. 18075039 10.1109/tbme.2007.908332

[81] Kuo B , McCallum RW , Koch KL , Sitrin MD , Wo JM , Chey WD , et al. Comparison of gastric emptying of a nondigestible capsule to a radio‐labelled meal in healthy and gastroparetic subjects. Aliment Pharmacol Ther. 2008;27(2):186–196. 17973643 10.1111/j.1365-2036.2007.03564.x

[82] Rao SS , Kuo B , McCallum RW , Chey WD , DiBaise J , Hasler WL , et al. Investigation of colonic and whole‐gut transit with wireless motility capsule and radiopaque markers in constipation. Clin Gastroenterol Hepatol. 2009;7(5):537–544. 19418602 10.1016/j.cgh.2009.01.017

[83] Maqbool S , Parkman HP , Friedenberg FK . Wireless capsule motility: comparison of the SmartPill GI monitoring system with scintigraphy for measuring whole gut transit. Dig Dis Sci. 2009;54(10):2167–2174. 19655250 10.1007/s10620-009-0899-9

[84] Filipe MI . Mucins and gastrointestinal malignancy. A new approach to the interpretation of biopsies. Acta Med Port. 1979;1(3):351–365. 551699

[85] Cummings JH , Macfarlane GT . The control and consequences of bacterial fermentation in the human colon. J Appl Bacteriol. 1991;70(6):443–459. 1938669 10.1111/j.1365-2672.1991.tb02739.x

[86] Gibson GR , Macfarlane GT , Cummings JH . Sulphate reducing bacteria and hydrogen metabolism in the human large intestine. Gut. 1993;34(4):437–439. 8491386 10.1136/gut.34.4.437 PMC1374298

[87] Mikolajczyk AE , Watson S , Surma BL , Rubin DT . Assessment of tandem measurements of pH and Total gut transit time in healthy volunteers. Clin Transl Gastroenterol. 2015;6:e100. 26158610 10.1038/ctg.2015.22 PMC4816255

[88] Farmer AD , Mohammed SD , Dukes GE , Scott SM , Hobson AR . Caecal pH is a biomarker of excessive colonic fermentation. World J Gastroenterol. 2014;20(17):5000–5007. 24803812 10.3748/wjg.v20.i17.5000 PMC4009533

[89] Farmer AD , Ruffle JK , Hobson AR . Linaclotide increases cecal pH, accelerates colonic transit, and increases colonic motility in irritable bowel syndrome with constipation. Neurogastroenterol Motil. 2019;31(2):e13492. 30353623 10.1111/nmo.13492

[90] Ringel‐Kulka T , Choi CH , Temas D , Kim A , Maier DM , Scott K , et al. Altered colonic bacterial fermentation as a potential pathophysiological factor in irritable bowel syndrome. Am J Gastroenterol. 2015;110(9):1339–1346. 26303129 10.1038/ajg.2015.220 PMC4983766

[91] Medtronic to discontinue SmartPill capsule [press release]. Online: GI & Hepatology News. [2023 June 22]. 2023.

[92] Asci C , Sharma A , Del‐Rio‐Ruiz R , Sonkusale S . Ingestible pH sensing device for gastrointestinal health monitoring based on thread‐based electrochemical sensors. Microchim Acta. 2023;190(10):385. 10.1007/s00604-023-05946-1 37698743

[93] Gopalakrishnan S , Thomas R , Sedaghat S , Krishnakumar A , Khan S , Meyer T , et al. Smart capsule for monitoring inflammation profile throughout the gastrointestinal tract. Biosens Bioelectron X. 2023;14:1–10. 10.1016/j.biosx.2023.100380 PMC10552446 37799507

[94] Kalantar‐Zadeh K , Berean KJ , Burgell RE , Muir JG , Gibson PR . Intestinal gases: influence on gut disorders and the role of dietary manipulations. Nat Rev Gastroenterol Hepatol. 2019;16(12):733–747. 31520080 10.1038/s41575-019-0193-z

[95] Kalantar‐Zadeh K , Berean KJ , Ha N , Chrimes AF , Xu K , Grando D , et al. A human pilot trial of ingestible electronic capsules capable of sensing different gases in the gut. Nat Electron. 2018;1(1):79–87.

[96] Ou JZ , Yao CK , Rotbart A , Muir JG , Gibson PR , Kalantar‐zadeh K . Human intestinal gas measurement systems: in vitro fermentation and gas capsules. Trends Biotechnol. 2015;33(4):208–213. 25772639 10.1016/j.tibtech.2015.02.002

[97] Zhou J , Berean K , Pedersen H , Thwaites PA , Gibson PR , Burgell R , et al. Ingestible gas‐sensing capsule for whole gut transit: prospective comparison with wireless pH‐motility capsule (SmartPill) in gastroparesis and chronic constipation. Gastroenterology. 2023;164(6), S–629.

[98] Shah A , Sahu S , Pederse H , Chrimes A , John J , Singh M , et al. Small intestinal bacterial overgrowth as assessed by an ingestible gas‐sensing device: a prospective comparison to both aspirate and breath testing. Gastroenterology. 2023;164:S55.

[99] Stine JM , Ruland KL , Beardslee LA , Levy JA , Abianeh H , Botasini S , et al. Miniaturized capsule system towards real‐time electrochemical detection of H2S in the gastrointestinal tract. Adv Healthc Mater. 2023:2302897. 10.1002/adhm.202302897 PMC11468942 38035728

[100] Mimee M , Nadeau P , Hayward A , Carim S , Flanagan S , Jerger L , et al. An ingestible bacterial‐electronic system to monitor gastrointestinal health. Science. 2018;360(6391):915–918. 29798884 10.1126/science.aas9315 PMC6430580

[101] Inda M , Jimenez M , Liu Q , Phan NV , Ahn J , Steiger C , et al. Ingestible capsule for detecting labile inflammatory biomarkers in situ. bioRxiv 2022:2022.2002.2016.480562.

[102] Singh S , Allan ND , Wahl C , Lee SN , Chuang E , Jones ML . Sa1717 – development of a swallowable diganostic capsule to monitor gastrointestinal health. Gastroenterology. 2019;156:S376.

[103] Rao SS , Moshiree B , Lee N , Jones ML , Chuang E , Singh S . S1282 Evaluation of smart capsule bacterial detection system (SCBDS) assay and duodenal culture in subjects suspected of SIBO and undergoing upper endoscopy: interim analysis. Am J Gastroenterol. 2020;115:S644.

[104] Hasler WL . The use of SmartPill for gastric monitoring. Expert Rev Gastroenterol Hepatol. 2014;8(6):587–600. 24881810 10.1586/17474124.2014.922869

[105] Kloetzer L , Chey WD , McCallum RW , Koch KL , Wo JM , Sitrin M , et al. Motility of the antroduodenum in healthy and gastroparetics characterized by wireless motility capsule. Neurogastroenterol Motil. 2010;22(5):527–533 e117. 20122128 10.1111/j.1365-2982.2010.01468.x

[106] Liao C‐H , Cheng C‐T , Chen C‐C , Jow UM , Chen CH , Lai YL , et al. An ingestible electronics for continuous and real‐time intraabdominal pressure monitoring. J Pers Med. 2020;11(1):12. 33374271 10.3390/jpm11010012 PMC7823632

[107] McKenzie JE , Osgood DW . Validation of a new telemetric core temperature monitor. J Therm Biol. 2004;29(7):605–611.

[108] Bongers C , Daanen HAM , Bogerd CP , Hopman MTE , Eijsvogels TMH . Validity, reliability, and inertia of four different temperature capsule systems. Med Sci Sports Exerc. 2018;50(1):169–175. 28816921 10.1249/MSS.0000000000001403

[109] Osterberg L , Blaschke T . Adherence to medication. N Engl J Med. 2005;353(5):487–497. 16079372 10.1056/NEJMra050100

[110] Hafezi H , Robertson TL , Moon GD , Au‐Yeung KY , Zdeblick MJ , Savage GM . An ingestible sensor for measuring medication adherence. IEEE Trans Biomed Eng. 2015;62(1):99–109. 25069107 10.1109/TBME.2014.2341272

[111] Otsuka and Proteus announce the first U.S . FDA apporval of a digital medicine system: Abilify MyCite (aripiprazole tablets with sensor). 2017; https://go.nature.com/2K1bro314

[112] Eisenberger U , Wüthrich RP , Bock A , Ambühl P , Steiger J , Intondi A , et al. Medication adherence assessment: high accuracy of the new ingestible sensor system in kidney transplants. Transplantation. 2013;96(3):245–250. 23823651 10.1097/TP.0b013e31829b7571 PMC3749815

[113] Belknap R , Weis S , Brookens A , Au‐Yeung KY , Moon G , DiCarlo L , et al. Feasibility of an ingestible sensor‐based system for monitoring adherence to tuberculosis therapy. PloS One. 2013;8(1):e53373. 23308203 10.1371/journal.pone.0053373 PMC3538759

[114] Weitschies W , Müller L , Grimm M , Koziolek M . Ingestible devices for studying the gastrointestinal physiology and their application in oral biopharmaceutics. Adv Drug Deliv Rev. 2021;176:113853. 34192551 10.1016/j.addr.2021.113853

[115] Parr AF , Sandefer EP , Wissel P , McCartney M , McClain C , Ryo UY , et al. Evaluation of the feasibility and use of a prototype remote drug delivery capsule (RDDC) for non‐invasive regional drug absorption studies in the GI tract of man and beagle dog. Pharm Res. 1999;16(2):266–271. 10100313 10.1023/a:1018884510163

[116] van der Schaar PJ , Dijksman JF , Broekhuizen‐de Gast H , Shimizu J , van Lelyveld N , Zou H , et al. A novel ingestible electronic drug delivery and monitoring device. Gastrointest Endosc. 2013;78(3):520–528. 23684148 10.1016/j.gie.2013.03.170

[117] Söderlind E , Abrahamsson B , Erlandsson F , Wanke C , Iordanov V , von Corswant C . Validation of the IntelliCap® system as a tool to evaluate extended release profiles in human GI tract using metoprolol as model drug. J Control Release. 2015;217:300–307. 26385166 10.1016/j.jconrel.2015.09.024

[118] Becker D , Zhang J , Heimbach T , Penland RC , Wanke C , Shimizu J , et al. Novel orally swallowable IntelliCap® device to quantify regional drug absorption in human GI tract using diltiazem as model drug. AAPS PharmSciTech. 2014;15:1490–1497. 25023947 10.1208/s12249-014-0172-1 PMC4245429

[119] Beccani M , Di Natali C , Aiello G , Benjamin C , Susilo E , Valdastri P . A magnetic drug delivery capsule based on a coil actuation mechanism. Proc Eng. 2015;120:53–56.

[120] Zheng L , Guo S , Kawanishi M . Magnetically controlled multifunctional capsule robot for dual‐drug delivery. IEEE Syst J. 2022;16(4):6413–6424.

[121] Abramson A , Caffarel‐Salvador E , Khang M , Dellal D , Silverstein D , Gao Y , et al. An ingestible self‐orienting system for oral delivery of macromolecules. Science. 2019;363(6427):611–615. 30733413 10.1126/science.aau2277 PMC6430586

[122] Abramson A , Caffarel‐Salvador E , Soares V , Minahan D , Tian RY , Lu X , et al. A luminal unfolding microneedle injector for oral delivery of macromolecules. Nat Med. 2019;25(10):1512–1518. 31591601 10.1038/s41591-019-0598-9 PMC7218658

[123] de Ávila BE , Angsantikul P , Li J , Angel Lopez‐Ramirez M , Ramírez‐Herrera DE , Thamphiwatana S , et al. Micromotor‐enabled active drug delivery for in vivo treatment of stomach infection. Nat Commun. 2017;8(1):272. 28814725 10.1038/s41467-017-00309-w PMC5559609

[124] Terry BS , Passernig AC , Hill ML , Schoen JA , Rentschler ME . Small intestine mucosal adhesivity to in vivo capsule robot materials. J Mech Behav Biomed Mater. 2012;15:24–32. 23026729 10.1016/j.jmbbm.2012.06.018

[125] Kong YL , Zou X , McCandler CA , Kirtane AR , Ning S , Zhou J , et al. 3D‐printed gastric resident electronics. Adv Mater Technol. 2019;4(3):1800490. 32010758 10.1002/admt.201800490 PMC6988123

[126] Bellinger AM , Jafari M , Grant TM , Zhang S , Slater HC , Wenger EA , et al. Oral, ultra‐long‐lasting drug delivery: application toward malaria elimination goals. Sci Transl Med. 2016;8(365):365ra157. 10.1126/scitranslmed.aag2374 PMC5264553 27856796

[127] Kirtane AR , Hua T , Hayward A , Bajpayee A , Wahane A , Lopes A , et al. A once‐a‐month oral contraceptive. Sci Transl Med. 2019;11(521):eaay2602. 31801885 10.1126/scitranslmed.aay2602

[128] Srinivasan SS , Alshareef A , Hwang AV , Kang Z , Kuosmanen J , Ishida K , et al. RoboCap: robotic mucus‐clearing capsule for enhanced drug delivery in the gastrointestinal tract. Sci Robot. 2022;7(70):eabp9066. 36170378 10.1126/scirobotics.abp9066 PMC10034646

[129] Mariello M , Eş I , Proctor CM . Soft and flexible bioelectronic micro‐systems for electronically controlled drug delivery. Adv Healthc Mater. 2023:e2302969. 10.1002/adhm.202302969 37924224

[130] Shalon D , Culver RN , Grembi JA , Folz J , Treit PV , Shi H , et al. Profiling the human intestinal environment under physiological conditions. Nature. 2023;617(7961):581–591. 37165188 10.1038/s41586-023-05989-7 PMC10191855

[131] Rees S , Ashton L , Iveson C , Chomka A , Thomsen S , Ng NH , et al. A device for sampling gastro‐intestinal material. Google Patents. 2019.

[132] Nejati S , Wang J , Sedaghat S , Balog NK , Long AM , Rivera UH , et al. Smart capsule for targeted proximal colon microbiome sampling. Acta Biomater. 2022;154:83–96. 36162763 10.1016/j.actbio.2022.09.050 PMC9986838

[133] Crosby WH , Army US , Kugler HW . Intraluminal biopsy of the small intestine. Am J Dig Dis. 1957;2(5):236–241. 13410880 10.1007/BF02231100

[134] Achkar E , Carey WD , Petras R , Sivak MV , Revta R . Comparison of suction capsule and endoscopic biopsy of small bowel mucosa. Gastrointest Endosc. 1986;32(4):278–281. 3743980 10.1016/s0016-5107(86)71846-4

[135] Research NF . MICRO‐study: The IntelliCap® System as a Gastrointestinal Fluid Sampling Tool (MICRO). 2016.

[136] Cui J , Zheng X , Hou W , Zhuang Y , Pi X , Yang J . The study of a remote‐controlled gastrointestinal drug delivery and sampling system. Telemed J E Health. 2008;14(7):715–719. 18817502 10.1089/tmj.2007.0118

[137] Finocchiaro M , Giosuè C , Drago G , Cibella F , Menciassi A , Sprovieri M , et al. Design of a magnetic actuation system for a microbiota‐collection ingestible capsule. Paper presented at: 2021 IEEE International Conference on Robotics and Automation (ICRA); 30 May‐5 June 2021; 2021.

[138] Kong K‐C , Cha J , Jeon D , Cho D . A rotational micro biopsy device for the capsule endoscope. Paper presented at: 2005 IEEE/RSJ International Conference on Intelligent Robots and Systems; 2005.

[139] Simi M , Gerboni G , Menciassi A , Valdastri P . Magnetic mechanism for wireless capsule biopsy. J Med Dev. 2012;6(1):017611.

[140] Yim S , Gultepe E , Gracias DH , Sitti M . Biopsy using a magnetic capsule endoscope carrying, releasing, and retrieving untethered microgrippers. IEEE Trans Biomed Eng. 2014;61(2):513–521. 24108454 10.1109/TBME.2013.2283369 PMC4023810

[141] Rehan M , Al‐Bahadly I , Thomas DG , Avci E . Development of a robotic capsule for in vivo sampling of gut microbiota. IEEE Robot Autom Lett. 2022;7(4):9517–9524.

[142] Soto F , Purcell E , Ozen MO , Sinawang PD , Wang J , Akin D , et al. Robotic pill for biomarker and fluid sampling in the gastrointestinal tract. Adv Intell Syst. 2022;4(6):2200030.

[143] Rao SSC , Quigley EMM , Chey WD , Sharma A , Lembo AJ . Randomized placebo‐controlled phase 3 trial of vibrating capsule for chronic constipation. Gastroenterology. 2023;164:1202–1210.e6. 36822371 10.1053/j.gastro.2023.02.013

[144] Ron Y , Halpern Z , Safadi R , Dickman R , Dekel R , Sperber AD . Safety and efficacy of the vibrating capsule, an innovative non‐pharmacological treatment modality for chronic constipation. Neurogastroenterol Motil. 2015;27(1):99–104. 25484196 10.1111/nmo.12485

[145] Nelson AD , Camilleri M , Acosta A , Boldingh A , Busciglio I , Burton D , et al. A single‐center, prospective, double‐blind, sham‐controlled, randomized study of the effect of a vibrating capsule on colonic transit in patients with chronic constipation. Neurogastroenterol Motil. 2017;29(7):1–6. 10.1111/nmo.13034 28177172

[146] Zhu JH , Qian YY , Pan J , He C , Lan Y , Chen WN , et al. Efficacy and safety of vibrating capsule for functional constipation (VICONS): a randomised, double‐blind, placebo‐controlled, multicenter trial. EClinicalMedicine. 2022;47:101407. 35518121 10.1016/j.eclinm.2022.101407 PMC9062239

[147] Abramson A , Dellal D , Kong YL , Zhou J , Gao Y , Collins J , et al. Ingestible transiently anchoring electronics for microstimulation and conductive signaling. Sci Adv. 2020;6(35):eaaz0127. 32923616 10.1126/sciadv.aaz0127 PMC7455191

[148] Ramadi KB , McRae JC , Selsing G , Su A , Fernandes R , Hickling M , et al. Bioinspired, ingestible electroceutical capsules for hunger‐regulating hormone modulation. Sci Robot. 2023;8(77):eade9676. 37099636 10.1126/scirobotics.ade9676 PMC10508349

[149] Do TN , Seah TET , Yu HK , Phee SJ . Development and testing of a magnetically actuated capsule endoscopy for obesity treatment. PloS One. 2016;11:1–23. 10.1371/journal.pone.0148035 PMC4729466 26815309

[150] Min J , Yang Y , Wu Z , Gao W . Robotics in the gut. Adv Therap. 2020;3(4):1900125.

[151] Valdastri P , Quaglia C , Susilo E , Menciassi A , Dario P , Ho C , et al. Wireless therapeutic endoscopic capsule: in vivo experiment. Endoscopy. 2008;40(12):979–982. 19065478 10.1055/s-0028-1103424

[152] MIT R. Ingestible Origami Robot . Robotics @ MIT Web site. https://robotics.mit.edu/ingestible‐origami‐robot. Published 2023. Updated 24/7/23. Accessed 24/7/2023; 2023.

[153] Vaccari M , Tudor T , Perteghella A . Costs associated with the management of waste from healthcare facilities: an analysis at national and site level. Waste Manag Res. 2018;36(1):39–47. 29132259 10.1177/0734242X17739968

[154] Saad RJ , Hasler WL . A technical review and clinical assessment of the wireless motility capsule. Gastroenterol Hepatol (N Y). 2011;7(12):795–804. 22347818 PMC3280411

[155] Rondonotti E , Herrerias JM , Pennazio M , Caunedo A , Mascarenhas‐Saraiva M , de Franchis R . Complications, limitations, and failures of capsule endoscopy: a review of 733 cases. Gastrointest Endosc. 2005;62(5):712–716. 16246685 10.1016/j.gie.2005.05.002

[156] Gravina R , Fortino G . Wearable body sensor networks: state‐of‐the‐art and research directions. IEEE Sens J. 2021;21(11):12511–12522.

[157] Klugman CM , Dunn LB , Schwartz J , Cohen IG . The ethics of smart pills and self‐acting devices: autonomy, truth‐telling, and trust at the dawn of digital medicine. Am J Bioeth. 2018;18(9):38–47. 10.1080/15265161.2018.1498933 30235091

[158] Matzeu G , Florea L , Diamond D . Advances in wearable chemical sensor design for monitoring biological fluids. Sens Actuat B Chem. 2015;211:403–418.

[159] Hasler WL , Rao SSC , McCallum RW , Krause RA , Nguyen LA , Schulman MI , et al. Influence of gastric emptying and gut transit testing on clinical management decisions in suspected gastroparesis. Clin Transl Gastroenterol. 2019;10(10):e00084. 31663906 10.14309/ctg.0000000000000084 PMC6919448

[160] Yu M . M2A™ capsule endoscopy: a breakthrough diagnostic tool for small intestine imaging. Gastroenterol Nurs. 2002;25(1):24–27. 11852828 10.1097/00001610-200201000-00007

[161] Cave D , Legnani P , Franchis R , Lewis B . ICCE consensus for capsule retention. Endoscopy. 2005;37:1065–1067. 16189792 10.1055/s-2005-870264

[162] Rezapour M , Amadi C , Gerson LB . Retention associated with video capsule endoscopy: systematic review and meta‐analysis. Gastrointest Endosc. 2017;85(6):1157–1168.e1152. 28069475 10.1016/j.gie.2016.12.024

[163] Lucendo AJ , Gonzalez‐Castillo S , Fernandez‐Fuente M , De Rezende LC . Tracheal aspiration of a capsule endoscope: a new case report and literature compilation of an increasingly reported complication. Dig Dis Sci. 2011;56(9):2758–2762. 21409372 10.1007/s10620-011-1666-2

[164] Yung DE , Plevris JN , Koulaouzidis A . Short article: aspiration of capsule endoscopes: a comprehensive review of the existing literature. Eur J Gastroenterol Hepatol. 2017;29(4):428–434. 28253209 10.1097/MEG.0000000000000821

